# Variability in Isotope Discrimination Factors in Coral Reef Fishes: Implications for Diet and Food Web Reconstruction

**DOI:** 10.1371/journal.pone.0013682

**Published:** 2010-10-27

**Authors:** Alex S. J. Wyatt, Anya M. Waite, Stuart Humphries

**Affiliations:** 1 The Oceans Institute, The University of Western Australia, Crawley, Australia; 2 School of Environmental Systems Engineering, The University of Western Australia, Crawley, Australia; 3 Department of Biological Sciences, University of Hull, Kingston-upon-Hull, United Kingdom; University of California Davis, United States of America

## Abstract

Interpretation of stable isotope ratios of carbon and nitrogen (δ^13^C and δ^15^N) is generally based on the assumption that with each trophic level there is a constant enrichment in the heavier isotope, leading to diet-tissue discrimination factors of 3.4‰ for ^15^N (ΔN) and ∼0.5‰ for ^13^C (ΔC). Diet-tissue discrimination factors determined from paired tissue and gut samples taken from 152 individuals from 26 fish species at Ningaloo Reef, Western Australia demonstrate a large amount of variability around constant values. While caution is necessary in using gut contents to represent diet due to the potential for high temporal variability, there were significant effects of trophic position and season that may also lead to variability in ΔN under natural conditions. Nitrogen enrichment increased significantly at higher trophic levels (higher tissue δ^15^N), with significantly higher ΔN in carnivorous species. Changes in diet led to significant changes in ΔN, but not tissue δ^15^N, between seasons for several species: *Acanthurus triostegus*, *Chromis viridis*, *Parupeneus signatus* and *Pomacentrus moluccensis*. These results confirm that the use of meta-analysis averages for ΔN is likely to be inappropriate for accurately determining diets and trophic relationships using tissue stable isotope ratios. Where feasible, discrimination factors should be directly quantified for each species and trophic link in question, acknowledging the potential for significant variation away from meta-analysis averages and, perhaps, controlled laboratory diets and conditions.

## Introduction

The analysis of stable isotope ratios of carbon and nitrogen (δ^13^C and δ^15^N) to determine an organism's diet and to reconstruct food webs is widespread, and δ^13^C and δ^15^N are increasingly being used in the study of coral reef trophodynamics. Interpretation of stable isotope ratios is generally based on the assumption that with each trophic level there is a constant enrichment in the heavier isotope, leading to diet-tissue discrimination factors of 3.4‰ for ^15^N (ΔN) and ∼0.5‰ for ^13^C (ΔC) [1]–[4]. Post [3] noted that, in his meta-analysis, discrimination did not vary significantly with organism body size, between species, functional groups or even habitats, but stressed that average discrimination factors can only be applied to entire food webs, with many multiple trophic pathways and species. In fact, it is increasingly being recognised that trophic discrimination has a high degree of variability around meta-analysis averages when examined for species or groups, e.g. [5]–[7]. Indeed, as Vander Zanden and Rasmussen [1] note, Minagawa and Wada's [2] value of 3.4‰ for ΔN was itself variable, being determined from the mean discrimination in only 16 individual estimates and with a standard deviation of 1.1‰. Similarly, Post's [3] value from 56 individual estimates had a standard deviation of 0.98‰. Despite this variability, many studies using isotope ratios must rely on assumed discrimination constants, often from different tissues or species, to make conclusions about diet or trophic position [6]. Many recent isotope studies of coral reef fishes apply meta-analysis average discrimination factors to the analysis of a single species or trophic group, e.g. [8]–[12].

While the mechanisms of diet-tissue discrimination are still not completely understood, tissue isotopes are generally accepted to be the result of a dynamic equilibrium between isotopic discrimination during assimilation and excretion [13]–[15]. Variability in discrimination is thus not surprising given the range of factors known to influence assimilatory and excretory processes within an organism. Tissue type, age or body size, diet quality, nutritional stress, feeding rate and excretory mechanisms are all known to influence discrimination [4]–[6], [15]–[19], and there can be substantial differences between organisms as a result [4]. The fact that feeding on mixed diets [20] and sample preparation [4], [7] can also significantly influence discrimination factors further argues against applying meta-analysis averages to focused field studies. Using incorrect discrimination constants, even though the error may be small, has the potential to lead to large errors in the estimation of food sources or trophic position [4], especially for ΔN and studies focused on few species or groups [3]. Consequently, for accurate estimates of diet or trophic position it is essential that discrimination factors be quantified, whether directly or by modelling e.g. [15], and variability accounted for in analyses (such as through Bayesian modelling, e.g. [21], [22]).

Despite the apparent variability and potential consequences, the extent to which changes in the many factors mentioned above can lead to differences in trophic discrimination within and between species in the field is not well quantified. In this study, discrimination factors are measured for variety of coral reef fishes at Ningaloo Reef, Western Australia and examined in the context of spatial and temporal factors that may lead to variation, and hence error in diet or trophic position estimates.

## Materials and Methods

### Site description & experimental overview

**Figure 1:**
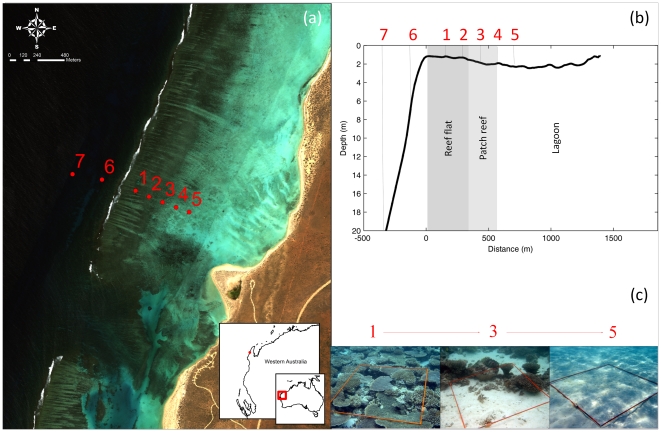
The study site, sampling stations and habitats at Sandy Bay, Ningaloo Reef, Western Australia. Panels show (a) sampling stations 1 to 7 across the reef (see Wyatt et al. [23] for more details on the location and hydrodynamics); (b) reef zonation and bathymetry between locations derived from hyperspectral imagery; and (c) representative images of the benthic habitat demonstrate the shift from the tabulate hard coral dominated reef flat (station 1), to patch reef (3) and sandy lagoon (5).

The study site at Sandy Bay, Ningaloo Reef, Western Australia is a typical fringing reef habitat. The site, including the prevailing hydrodynamics, is described in detail in Wyatt et al. [23]. During May 2007 and May and Nov 2008 a total of 152 individual fish specimens were collected with line or spear under the approval of the Department of Environment and Conservation (permit numbers SF006335, CE001989 and SW012041). Reef-based fishes were collected from one of seven sites that traverse the reef, from reef slope to lagoon (Figure 1a), while pelagic species were caught by trolling along the reef slope in the vicinity of stations 6 and 7. Upon collection specimens were immediately placed on ice and transported to shore for dissection (maximum time between collection and dissection was 3 hrs).

### Sampling and analysis procedures

Paired tissue and gut samples were taken from each specimen for isotopic analysis. Tissue samples were dissected from white muscle near the base of the tail, while gut samples were collected, where possible, from the anterior alimentary canal (very small specimens often precluded separating fore and hind gut contents) and placed onto Whatman GF/F filters. Samples were stored frozen (−20°C) until analysis within three months. Tissue samples were dried at 60°C for 48 hrs before being ground to a powder using a Retsch® ball mill (Haan, Germany). Gut samples on GF/F filters were dried for 24 hrs and then acidified dropwise using 1 N HCl to remove carbonaceous material, before being re-dried for 24 hrs. This method of acidifying filters (direct, no rinsing) has been found to be the most effective method of removing the influence of carbonate on δ^13^C without significantly altering δ^15^N (A.S.J.W., unpublished data).

Samples were combusted to N_2_ and CO_2_ in tin capsules (12×5 mm, SerCon, Cheshire, UK) using an elemental analyser (ANCA-GSL, Europa Scientific Ltd., Crewe, UK). The N_2_ and CO_2_ were purified by gas chromatography and the nitrogen and carbon elemental composition and isotope ratios determined by continuous flow isotope ratio mass spectrometry (20-20 IRMS, Europa Scientific Ltd., Crewe, UK). Reference materials of known elemental composition and isotopic ratios were interspaced with the samples for calibration (USGS40, δ^15^N = −4.52‰, δ^13^C = −26.39‰; USGS41, δ^15^N = 47.57‰, δ^13^C = 37.63‰). Raw nitrogen and carbon elemental composition and isotope ratio data were corrected for instrument drift and blank contribution using ANCA-NT software (Europa Scientific Ltd., Crewe, United Kingdom). Nitrogen isotope ratios (δ^15^N) are reported in parts per thousand (per mil, ‰) relative to N_2_ in air and carbon isotope ratios (δ^13^C ) relative to Pee Dee Belemnite (V-PDB) according to the following formula:
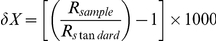
where X is ^15^N or ^13^C and R is the ratio of heavy to light isotope (^15^N∶^14^N or ^13^C∶^12^C). Repeatability for δ^15^N was ±0.17‰ and δ^13^C±0.12‰.

Diet-tissue discrimination factors were determined by subtracting the gut isotope value from the tissue value for each specimen:
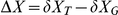
where X is ^15^N or ^13^C for tissue (T) and gut (G).

### Statistical analysis

Statistical analysis was performed in SPSS v17.0. A general linear model analysis of variance (ANOVA) was used to check for significant differences between groups after confirming homogeneity of variance using Levene's Test. Post-hoc differences were examined with Fisher's LSD.

## Results

**Figure 2:**
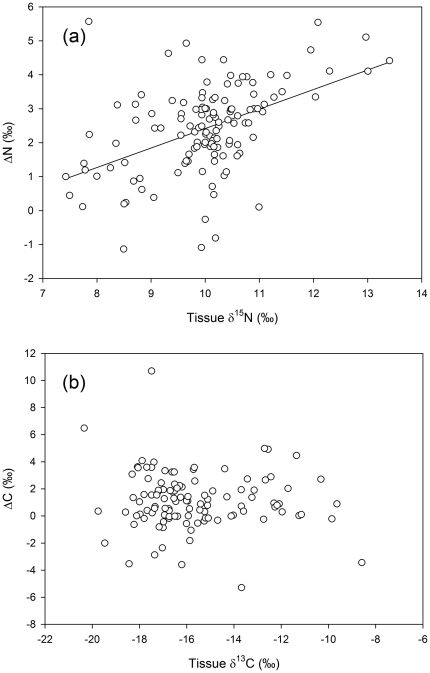
Fish tissue isotopes compared to gut content isotopes. Data shows isotope ratios at the time of sampling for (a) nitrogen (δ^15^N, n = 126) and (b) carbon (δ^13^C, n = 111). Solid lines represents significant linear regression relationships for nitrogen (Tissue δ^15^N = 0.325×Gut δ^15^N+7.535; r^2^ = 0.138; F_[1,124]_ = 19.93, p<0.001) and carbon (Tissue δ^13^C = 0.547×Gut δ^13^C−6.472; r^2^ = 0.583; F_[1,109]_ = 152.2, p<0.001).

Nitrogen discrimination factors (ΔN) were obtained for 126 of the 152 individuals sampled, covering 24 species and four trophic groups (Table 1). Due to instrument overloading, carbon discrimination factors (ΔC) were only obtained for 111 individuals from 22 species. Amongst these individuals there was evidence of marked deviation in both ΔN and ΔC away from published constants. Few species displayed average ΔN close to 3.4‰ [2], [3], with a study average of 2.4‰ and a wide individual range, −1.1 to 5.6‰ (Table 1). Carbon discrimination was also widely variable, ΔC ranging from −5.3 to 11, with a study average of 1.1‰. There was a significant relationship between the isotope ratios of an individual's tissue and its gut contents at the time of sampling, for both δ^15^N and δ^13^C (r^2^ = 0.138, p<0.001 and r^2^ = 0.583, p<0.001, respectively; Figure 2).

**Table 1:** Average tissue δ^15^N, ΔN between tissue and gut δ^15^N, tissue δ^13^C, and ΔC between tissue and gut δ^13^C (all in ‰) for individuals from 26 species, showing stations and number collected (n).

Species	Common name	Stations (n)	δ^15^N (n)	ΔN (n)	δ^13^C (n)	ΔC (n)
**Herbivores**						
*Acanthurus triostegus*	Convict surgeonfish	1 (12), 5 (2), 6 (1)	8.39±0.1 (23)	1.67±0.4 (15)	−12.85±0.4 (23)	1.32±0.3 (15)
*Chrysiptera unimaculata*	Onespot demoislle	1 (1), 2 (1), 3 (1), 6 (9)	9.78±0.1 (13)	1.94±0.3 (12)	−16.18±0.4 (13)	0.22±0.4 (10)
*Stegastes fasciolatus*	Pacific gregory	1 (3), 3 (7), 6 (14), 7 (4)	10.00±0.1 (31)	2.49±0.2 (28)	−16.23±0.6 (31)	1.53±0.4 (25)
*Stegastes nigricans*	Dusky gregory	6 (1), 3(1)	10.08±0.1 (2)	1.86±1.1 (2)	−16.24±0.7 (2)	−1.05±1.8 (2)
**Planktivores**						
*Abudefduf sexfasciatus*	Scissortail sergeant	1 (5), 6 (7)	10.57±0.1 (15)	3.22±0.2 (12)	−16.67±1.0 (15)	2.21±1.0 (11)
*Chromis cinerascens*	Green puller	6 (1)	10.91±0.1 (2)	3.00±0.0 (1)	−18.67±0.0 (2)	n.d. (0)
*Chromis viridis*	Blue-green damselfish	1 (7), 3 (1)	10.14±0.1 (11)	1.63±0.4 (8)	−17.30±0.3 (11)	0.93±0.3 (8)
*Dascyllus aruanus*	Humbug dascyllus	1 (2), 4 (1)	10.18±0.2 (3)	2.33±0.3 (3)	−15.01±0.9 (3)	2.09±0.3 (2)
*Dascyllus reticulatus*	Reticulate dascyllus	1 (1), 7 (2)	10.58±0.5 (3)	3.22±0.5 (3)	−17.02±2.3 (3)	2.51±1.3 (3)
*Dascyllus trimaculatus*	Three-spot dascyllus	1 (2), 6 (1)	10.90±0.2 (5)	2.31±0.3 (3)	−16.51±0.1 (5)	0.75±0.8 (2)
*Pomacentrus albicaudatus*	Whitefin damsel	6 (1)	10.17 (1)	1.65 (1)	−14.76 (1)	n.d. (0)
*Pomacentrus chrysurus*	Whitetail damsel	6 (2)	11.18±0.1 (2)	3.24±0.1 (2)	−18.60±0.0 (2)	−3.54±0.0 (1)
*Pomacentrus coelestis*	Neon damsel	1 (3)	10.14±0.1 (3)	0.80±0.9 (3)	−18.61±0.2 (3)	−0.88±0.6 (3)
*Pomacentrus moluccensis*	Lemon damsel	1 (8)	10.13±0.1 (9)	3.08±0.3 (8)	−16.29±0.3 (9)	0.22±0.3 (6)
*Pterocaesio tile*	Neon fusilier	6 (3)	10.15±0.1 (3)	2.30±0.2 (3)	−17.25±0.6 (3)	1.40±0.2 (3)
**Carnivores**						
*Cephalopholis sexmaculata*	Sixblotch hind	5 (1)	9.94 (1)	2.49 (1)	−10.32 (1)	2.71 (1)
*Lethrinus miniatus*	Trumpet emperor	7 (2)	13.19±0.2 (2)	4.76±0.3 (2)	−16.43±0.0 (2)	0.99±0.4 (2)
*Lethrinus nebulosus*	Spangled emperor	5 (2), 7(2)	11.15±0.8 (4)	3.29±0.3 (4)	−12.63±2.0 (4)	−2.27±0.8 (4)
*Lutjanus sebae*	Emperor red snapper	7 (1)	13.55 (1)	n.d. (0)	−16.56 (1)	n.d. (0)
*Parapercis clathrata*	False-eye grubfish	3 (1)	10.62 (1)	n.d. (0)	−10.26 (1)	n.d. (0)
*Parupeneus signatus*	Black-spot goatfish	3 (4), 6 (1), 7 (2)	9.66±0.4 (7)	2.19±0.6 (7)	−13.16±1.7 (7)	1.51±0.6 (5)
*Pristipomoides filamentosus*	Crimson jobfish	7 (2)	12.19±0.1 (2)	4.83±0.7 (2)	−17.81±0.8 (2)	0.96±0.4 (2)
*Sarda orientalis*	Striped bonito	6–7 (1)	11.51 (1)	3.98 (1)	−17.80 (1)	−0.19 (1)
*Thunnus tonggol*	Longtail tuna	6–7 (2)	11.61±0.3 (2)	4.73 (1)	−16.90±0.0 (2)	3.55 (1)
**Detritivores**						
*Amblygobius phalaena*	Banded goby	5 (2)	7.47±0.0 (3)	0.72±0.3 (2)	−12.48±0.8 (3)	4.72±0.3 (2)
*Gobiodon histrio*	Broad-barred maori goby	4 (2)	8.12±0.1 (2)	1.13±0.1 (2)	−12.55±0.0 (2)	2.77±0.1 (2)
**Average** **(range)**			**9.96** **(7.42–13.6)**	**2.41** **(−1.14–5.57)**	**−15.6** **(−20.3–−8.59)**	**1.13** **(−5.29–10.70)**

Common names and trophic group based on Froese & Pauly [39]. Data are mean ± s.e and show the number of individuals each calculation is based on (n). n.d. = no data.

### ΔN

**Figure 3:**
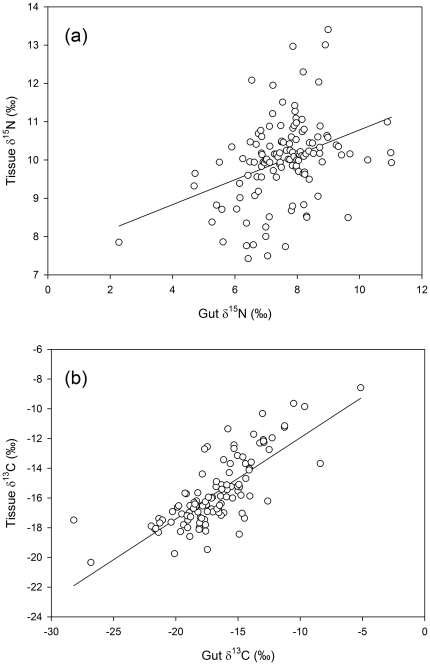
Discrimination factors versus tissue isotopic composition. Data is shown for (a) nitrogen (ΔN vs. δ^15^N, n = 126) and (b) carbon (ΔC vs. δ^13^C, n = 111 ). Solid line represents a significant linear regression relationship for nitrogen (ΔN = 0.574×δ^15^N–3.32; r^2^ = 0.226; F_[1,124]_ = 36.23, p<0.001). There was no relationship for carbon (r^2^ = 0.005; F_[1,109]_ = 0.0569, p = 0.452).

A proportion (23%) of the variation in nitrogen discrimination across the study can be explained by variation in tissue δ^15^N, with ΔN increasing significantly with increasing trophic level (as indicated by tissue δ^15^N, r^2^ = 0.226; F_[1,124]_ = 36.23, p<0.001, Figure 3a).

**Figure 4:**
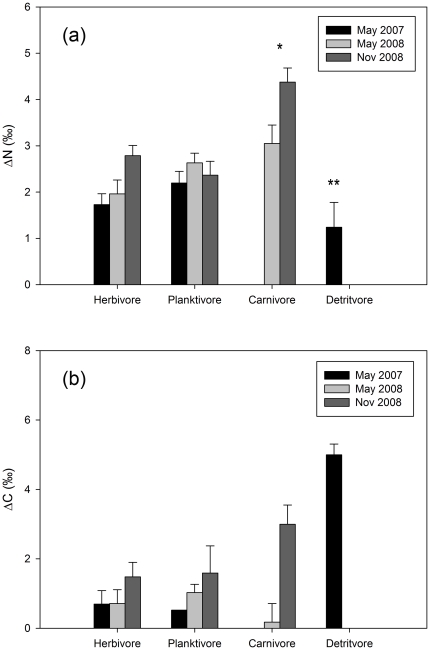
Discrimination factors by trophic group. Data is shown for herbivorous, planktivorous, carnivorous and detritivorous trophic groups in terms of (a) nitrogen (ΔN) and (b) carbon (ΔC). Data are mean ± s.e. (n as per Table 1); * and ** denote significantly different trophic groups (no significant seasonal differences or season×trophic group interactions).

Trophic groups had significantly different ΔN (F_[1,117]_ = 4.192, p<0.01, Figure 4a). Despite some qualitative evidence of seasonal differences within the trophic groups, there were no significant seasonal differences, or interaction, in the season×trophic group ANOVA model (but see seasonal difference for individual species below). Averaged across seasons, carnivores had higher ΔN than other groups and detritivores lower, with herbivores and planktivores not significantly different to each other.

Seasonal and spatial aspects of intra-species variation in discrimination are difficult to comprehensively address. This experiment was designed in a balanced way, so that the same fish species would be sampled in each season at a number of locations. Unfortunately, due to the fact that many coral reef species are associated with different zones, it was not always possible to find the same target species at different sites. Further, the loss of data due to instrument overloading decreased the number of replicate samples. As such, season and location as factors in changes in trophic discrimination can only be examined for a selected number of species for which data could be obtained in different seasons and/or locations.

**Figure 5:**
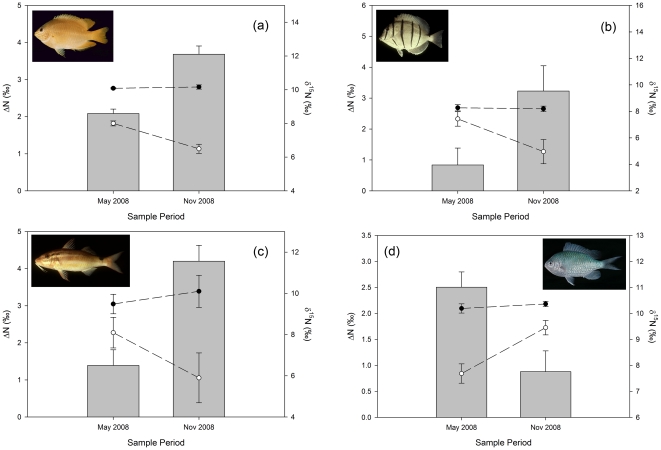
Seasonal variation in isotope discrimination factors. Plots show average nitrogen discrimination factors (ΔN, bars, left axis), tissue δ^15^N (solid circle, right axis) and gut δ^15^N (hollow circle, right axis) during May and Nov 2008 for (a) *Pomacentrus moluccensis* and (b) *Acanthurus triostegus* from 1 and 6a; (c) *Parupeneus signatus* sampled from 2, 3, 4, 6 and 7; and (d) *Chromis viridis* sampled from station 1. All ΔN and gut δ^15^N differences significant, no significant tissue δ^15^N differences or sample period×location interactions. See Figure 1 for locations. Fish images obtained from Froese & Pauly [39]; copyright J.E. Randall, 1997, used with permission. Data are mean ± s.e. (*n* as per Table 1).

There was preliminary evidence of significant seasonal differences in nitrogen discrimination for four species (67% of species with seasonally replicated samples). Nitrogen discrimination was significantly lower in May 2008 compared to Nov 2008 for *Pomacentrus moluccensis* (F_[1,6]_ = 26.08, p<0.01, Figure 5a), *Acanthurus triostegus* (F_[1,7]_ = 7.404, p<0.05, Figure 5b) and *Parupeneus signatus* (F_[1,2]_ = 21.66, p<0.05, Figure 5c) and. In contrast, *Chromis viridis* sampled had significantly higher discrimination in May 2008 (F_[1,4]_ = 12.10, p<0.05, Figure 5d). Increased ΔN in Nov was accompanied by lower gut δ^15^N in *P. moluccensis* (F_[1,6]_ = 15.99, p<0.01, Figure 5a), *A. triostegus* (F_[1,7]_ = 7.418, p<0.05, Figure 5b) and *P. signatus* (F_[1,2]_ = 33.98, p<0.05, Figure 5c). In contrast, *C. viridis* had significantly higher gut δ^15^N in Nov (F_[1,4]_ = 15.12, p<0.05, Figure 5d). There was no evidence of any significant location effects on ΔN, or season×location interactions, for any of the eleven species sampled at multiple locations and seasons.

### ΔC

In contrast to ΔN, there was little evidence of significant inter- and intra-species variation in ΔC. There was no significant relationship between δ^13^C and ΔC (Figure 3b), no significant trophic group or seasonal differences, or interactions (Figure 4b), and no significant changes in ΔC for any of the eleven species sampled at multiple locations and seasons.

There was also no significant relationship between discrimination factors (ΔN or ΔC) and either tissue or gut C∶N ratios. Variations in C∶N ratios only explained a very small proportion (<5%) of the variation in discrimination factors.

## Discussion

This study is the first to attempt to quantify isotope discrimination factors in situ for a variety of fish species across multiple trophic levels. Although the large amount of variability in discrimination factors documented in the study is in part a consequence of conducting the study under inherently variable field conditions and using gut contents as a dietary proxy, the results indicate that caution is necessary in applying discrimination factors to isotope data gathered from natural populations. There is the potential for significant variation in discrimination factors away from meta-analysis averages or those determined under controlled laboratory conditions.

### Discrimination in the gut and between tissues

A significant potential source of variability in diet-tissue discrimination factors measured in this study was the use of gut contents to represent an individual's diet. While tissue samples represent integration over time with respect to nutrient input (e.g. weeks to months [24]), gut samples represent a ‘snap shot’ of diet [25], containing only material that the specimen ingested immediately before collection (e.g. hours). Thus, in addition to containing material that will be excreted rather than incorporated into the fish's tissue, the gut may also contain an atypical diet at the time of sampling or significant amounts of non-dietary material, i.e. ingestion does not imply assimilation into tissue. This kind of variation in gut contents almost certainly contributed in part to the large variation in discrimination factors measured. Differential assimilation of different components of the diet can also mean that tissues reflect the isotopic composition of particular nutrient components from which they are synthesised, rather than the bulk diet [20], [26], [27]. There was however a significant relationship between the isotope ratios of an individual's tissue and its gut contents at the time of sampling, for both δ^15^N and δ^13^C, suggesting that, in general, the gut samples were a reasonable representation of the temporally averaged diet assimilated into tissues. Future work should consider investigating the role of differential assimilation of dietary components in driving discrimination factor variation, such as though the use of compound-specific isotope analysis.

There is also the potential for gut contents to be isotopically different to diet due to alteration during ingestion and digestion. Few studies have examined diet-gut isotope differences in fishes, which requires that fishes are fed a controlled diet of known isotopic composition under laboratory conditions. One of the few studies examining isotopic alteration of gut contents relative to diet during both ingestion and digestion by Guelinckx et al. [14] suggested that alteration is likely to be small compared to the total discrimination between diet and tissue, especially where fore-gut contents are analysed. Changes in the δ^15^N of diet in the fore gut of *Pomatoschistus minutus* were small relative to overall discrimination (diet to tissue) and negligble for δ^13^C. Diet to fore gut discrimination (i.e. largely undigested material) led to enrichment in δ^15^N by 1.31‰ (approximately 20% of the total ΔN of 6.6‰), with no signficant difference in δ^13^C. Discrimination during digestion (i.e. diet compared to hindgut/faecal material), has been investigated in more detail but without consensus. Results have ranged from depletion [28], to no change [29] to enrichment [14], [30]. Guelinckx et al. [14] found that digestive alteration of gut contents, together with the additon of excretory products, represented approximately 9% and 12%, respectively, of the total diet to tissue discrimination observed in *P. minutus* (6.6‰ for ΔN and 6.66‰ for ΔC). While efforts were made in the current study to sample fore gut contents from each specimen, it was very difficult to exclude hind gut contents especially for very small species. Inclusion of mixed gut contents is likely to alter the discrimination measured, since hind gut contents are generally composed increasingly of excretory material that is significantly different isotopically to the diet [14].

Although it is not possible to definitively determine diet to gut content isotope changes in the field, since the diet is not known, our data tend to support a minimal amount of discrimination between the diet and gut. As an example, the average gut δ^15^N of 5.99‰ (±0.13, s.e.) for *Abudefduf sexfasciatus* was very similar to the of δ^15^N of their assumed zooplankton prey at around 6‰ (6.19‰ (±0.01) for the >500 µm fraction, 6.09‰ (±0.00) for >300 µm and 6.26‰ (±0.20) for >105 µm, A.S.J. Wyatt, unpublished data). Thus any discrimination between diet and gut contents would appear to be negligible compared to the total discrimination between diet/gut and tissue (ΔN of 3.22 (±0.2) for *Abudefduf sexfasciatus*, Table 1). This would appear to support Guelinckx et al.'s [14] suggestion that the timing of sampling after feeding time, and hence the degree of digestion, is relatively unimportant and does not confound isotopic values for diet determined from gut contents. Although gut content analysis is very difficult for small amounts of gut contents subject to differing degrees of digestion, future more focused studies should consider gut content analysis as a means of directly quantifying dietary components and the role of discrimination during digestion.

Variations in tissue composition may also lead to observations of variable discrimination factors. Isotopic composition is known to vary significantly between different tissue types, which in turn vary in composition over different temporal scales [19], [31], [32]. In this study only white muscle tissue was examined for tissue isotope analysis and should therefore represent similar metabolic processes and rates between samples. However, the carbon isotope composition of fish tissue is known to change depending on lipid content because lipids are ^13^C-depleted relative to proteins [33]–[35]. Lipid extraction significantly alters δ^15^N, e.g. [34] but see [36], and was not considered suitable in this study where δ^15^N and δ^13^C were obtained from a single sample. Further, a meta-analysis by Caut et al. [6] did not reveal a significant effect of lipid extraction on discrimination factors. Regardless, any potential effect of variation in lipid content between samples would be confined to our estimates of ΔC, which showed no significant differences between sample groups.

### Inter-species differences in discrimination factors

The limitations of gut contents for representing diet aside, there are a number of additional factors that could explain the wide variations in discrimination factors within and between species. Differences in discrimination factors between species are expected due to differences in diet and/or metabolic processes. For instance, Mill et al. [16] suggested that herbivorous fishes often have markedly higher ΔN than the meta-analysis averages of 3.4‰. This could be due to differences in the diet quality (C∶N ratio) of herbivorous relative to carnivorous species (the latter displaying much more consistent nitrogen discrimination [16]) and/or metabolic differences (such as herbivorous fish having greater excretion rates [16]). In contrast, other studies have found that carnivorous species have significantly higher ΔN, attributed to a high protein diet [1], [4]. In the current study, fish species were sampled across a range of trophic levels, with δ^15^N ranging from 7.4 to 14‰ (Table 1). The species sampled therefore represent a range of trophic groups expected to have widely different diets, including herbivores, planktivores (e.g. zooplankton), carnivores (e.g. benthic invertebrates and other fishes), and detritivores. Indeed, a proportion (23%) of the variation in nitrogen discrimination across the study was explained by variation in tissue δ^15^N, with ΔN increasing significantly with increasing trophic level. There were also significant differences in ΔN between the trophic groups. Thus, in contrast to the findings of Mill et al [16], herbivores did not display significantly higher ΔN as would be expected a priori based on differences in diet quality (Figure 4a). In fact, diet quality appeared to have little influence on discrimination factors for any trophic groups in this study, with no significant relationships between discrimination factors and either tissue or gut C∶N, suggesting that factors other than diet quality led to differences in discrimination between trophic groups.

### Intra-species differences in discrimination factors

Variation in diet-tissue discrimination could also be expected at the intra-species level due to differences in diet, feeding rate and assimilatory and excretory mechanisms between individuals. Such differences could reflect the life history stage of the individual, as well as having spatial (e.g. habitat) and seasonal components. Ontogenetic changes in diet, as well as metabolism, have previously been demonstrated in coral reef fishes, leading to differences in tissue isotope composition for differently aged organisms of the same species, e.g. [37]. Thus it could be expected that the degree of discrimination between tissue and diet would also change with size as diet and metabolism changes. No attempt was made in this study to examine changes in discrimination with fish size, however samples of a single species were targeted so that they were all of a similar size and within the adult size range for that species. Thus, ontogenetic changes in diet and trophic discrimination are unlikely to explain the intra-species variability observed.

Two other factors are possible in driving the intra-species seasonal changes in ΔN observed: changes in metabolism driven by reproductive cycles or environmental change, or changes in diet. Ningaloo Reef is influenced by distinctly seasonal oceanographic conditions that alter both physical conditions on the reef and the biogeochemical environment [23]. There is the possibility that reproductive cycles or metabolic changes, for instance due to significantly cooler water temperatures in November than May (average 24°C compared to 28°C, A.S.J. Wyatt et al., unpublished data), led to seasonal changes in ΔN, e.g. [5]. However, despite a lack of evidence for significant seasonal change in diet from tissue δ^15^N, gut δ^15^N suggests that a change in diet may have been the principal factor in the altered discrimination factors between seasons for all species with sufficient replication for temporal analysis (although the small sample numbers mean these data should still be viewed as preliminary). The significant increases or decreases in δ^15^N of gut samples for each species mirrored the direction of change in ΔN and were of similar magnitude (Figure 5). Increased ΔN in Nov appeared to be driven by lower gut δ^15^N in *P. moluccensis*, *P. signatus* and *A. triostegus*. Interestingly *C. viridis*, also a planktivore and therefore expected to have a similar diet to *P. moluccensis*, had significantly higher gut δ^15^N in Nov. Indeed, tissue δ^15^N suggests these species have similar diets over time, averaging 10.1 and 10.3‰ for *C. viridis* and *P. moluccensis*, respectively, so the differences in seasonal change is puzzling and may warrant more detailed investigation.

The seasonally changing diets appeared to alter gut isotope composition but not tissue composition. There are several potential explanations for this. Firstly, the changes may simply be a reflection of the lag time between the isotope compositions of the diet and tissues changing. The isotopic composition of a tissue changes in response to diet through two mechanisms: dilution – the formation of new tissue with the new dietary composition, and metabolic turnover – the replacement of old tissue with new during tissue repair [19], [31], [38]. Thus, even with significant dietary change, a significant change in tissue δ^15^N may not be detected if there has not been sufficient time for marked dilution or turnover. The consistency of measurements within periods, which represent sampling over 3–6 weeks, and the time between the two periods (approximately six months) suggests that there should have been sufficient time for tissue composition to reflect the diet change.

Secondly, the seasonal change in discrimination could be due to the composition of the diet. There was no evidence of a significant change in diet quality (C∶N ratio of gut contents) for any of the species that could explain altered discrimination. Feeding on a similar composition prey at a different trophic level could also be expected to alter discrimination. The gut isotopes of *P. moluccensis*, *P. signatus* and *A. triostegus*, suggest that they were feeding almost a trophic level lower (average gut δ^15^N decreased ∼1.5–2.5‰) when discrimination was higher, and conversely for *C. viridis* when discrimination decreased. This is however contrary to the general increase in discrimination with increasing trophic level (Figure 3a), and requires further investigation.

In contrast to apparently strong seasonal effects on discrimination, there was little evidence of any changes with location. The lack of location effects may be partially due to the fact that many species are mobile and move between feeding sites, thereby integrating any factors likely to affect discrimination. For instance, there was no significant difference in *Abudefduf sexfasciatus* ΔN between stations 1 and 6, but it is likely that there is exchange of fish at these two nearby stations, with large populations congregating and feeding between the reef slope and forward reef flat. However, *Stegastes fasciolatus* (a more site specific species) also showed no location changes in ΔN, even though it was sampled across the reef (stations and numbers as per Table 1), in vastly different habitats ranging from reef slope (20 m water depth) to the shallow reef flat (2 m), and displayed some evidence of changing diet (A.S.J.W., unpublished data). The presumption must therefore be that no diet or metabolic factors changed sufficiently to alter discrimination for *S. fasciolatus* across this range.

### Implications of discrimination factor variation

The variations in ΔN presented above, ranging from trophic group to intra-species level differences, confirm that meta-analysis averages for ΔN are likely to be unsuitable for examining diets in a small number of species or a limited number of trophic links under field conditions [3], [7]. For instance, the application of meta-analysis average diet-tissue discrimination factors to tissue δ^15^N would mask the apparent species-specific seasonal dietary shifts observed through quantification of ΔN in the four species above. While habitat seemed to have little influence on ΔN, seasonality in ΔN variation was highly species-specific and requires further examination in future studies if isotope values are to be accurately interpreted. In contrast, ΔC, although variable, did not appear to be significantly influenced by trophic level or group, by season, or by location. Thus, meta-analysis averages may indeed be more applicable in the case of ΔC [3]. The direct quantification of discrimination factors may be especially important with the increasing use of Bayesian mixing models that allow uncertainty in discrimination factors to propagate through the analysis, returning a true probability distribution of estimated dietary proportions, e.g. [21], [22]. Such analysis is dependent on measuring and understanding discrimination factor variation. Furthermore, small differences in discrimination factors also contain important information on the feeding rate and metabolic state of individuals [15], and thus may warrant investigation independent of their influence on food web and trophic position analyses.

### Conclusions

Although the use of gut contents to represent diet requires some caution, the results of this study confirm that, where feasible, discrimination factors should be directly quantified for each species and trophic link in question, acknowledging the potential for significant variation away from meta-analysis and controlled laboratory averages under variable field conditions. Future studies examining the trophic ecology of fishes at the species level would be greatly enhanced by detailed data on the variability in discrimination factors, ideally obtained from a large number of individuals over space and time. The addition of tissue and dietary-component specific analysis, such as through compound-specific isotope analysis, is likely to greatly enhance our understanding of the processes influencing discrimination factor variation, and thereby the applicability of stable isotope analyses to trophic ecology.
